# Women's strategies for managing domestic violence during pregnancy: a qualitative study in Iran

**DOI:** 10.1186/s12978-021-01276-8

**Published:** 2022-03-02

**Authors:** Malikeh Amel Barez, Raheleh Babazadeh, Robab Latifnejad Roudsari, Mojtaba Mousavi Bazaz, Khadigeh Mirzaii Najmabadi

**Affiliations:** 1 Student Research Committee, School of Nursing and Midwifery, Mashhad University of Medical Sciences, Mashhad, Iran; 2 Nursing and Midwifery Care Research Center, Mashhad University of Medical Sciences, Mashhad, Iran; 3 Department of Midwifery, School of Nursing and Midwifery, Mashhad University of Medical Sciences, Mashhad, Iran; 4 Department of Community Medicine, Faculty of Medicine, Mashhad University of Medical Sciences, Mashhad, Iran

**Keywords:** Domestic violence, Pregnancy, Coping strategy, Qualitative study

## Abstract

**Background:**

Domestic violence during pregnancy is a severe public health problem. Abused pregnant women are confronted with the threats posed by domestic violence. Pregnancy and protection of the unborn child could affect maternal strategies for managing violence. The purpose of this study was to explore Iranian women's strategies for managing domestic violence during pregnancy.

**Methods:**

This qualitative study was conducted in October 2019 to June 2021 in Mashhad, Iran. Data were collected through individual semi structured interviews with 13 women who experienced perinatal domestic violence, two relatives and 24 related specialists as well as two focus group discussions with attendance of 20 abused mothers until the data saturation was achieved. Data were analyzed by the conventional content analysis approach of Graneheim and Lundman.

**Results:**

The main themes "escape strategies" and "situation improvement strategies" were emerged as the result of data analysis. Escape strategies was comprised of three categories including concealment, passive dysfunctional behaviors and neutral behaviors to control maternal emotional distress. Situation improvement strategies was comprised of three categories including active self-regulation, protecting family privacy and help seeking to control violence.

**Conclusion:**

Understanding the experience of managing domestic violence among pregnant women is essential to design evidence based violence prevention programs, which enable supportive healthcare and social systems to encourage abused mothers to use more effective strategies and seeking help to overcome domestic violence.

## Plain Language Summary

Domestic violence during pregnancy threatens the health of the mother and the fetus. Mothers who experience domestic violence often use several strategies to decrease the violence. This study sought women's experiences in managing domestic violence during pregnancy. 13 women who experienced perinatal domestic violence, one daughter, one husband and 24 specialists as well as two focus group discussions with attendance of 20 abused mothers were interviewed in Mashhad in the north east of Iran. The results showed abused mothers used escape strategies and situation improvement strategies for managing domestic violence. Mostly abused mothers used emotion oriented strategies such as escape strategies for managing violence. These strategies included of concealment, passive dysfunctional behaviors and neutral behaviors. Some mothers with regard to individual decision-making and individual abilities tried to improve their situation by active self-regulation, protecting family privacy and help seeking. The results of these various strategies vary from reducing domestic violence to not changing the intensity of the violence or intensifying the perinatal domestic violence. In conclusion: inappropriate strategies against domestic violence could be threatening for fetal and maternal safety and appropriate strategies should be considered in perinatal education. Understanding the experience of managing domestic violence among abused pregnant women is essential to design evidence based domestic violence prevention strategies and programs.

## Introduction

Domestic violence is a public health concern and human rights violation affecting more than one third of all women in global [1]. Domestic violence and intimate partner violence are frequently used interchangeably in the literature [2]. It is estimated that one in three women disclose domestic violence during or after pregnancy, but prevalence differs depending on the location [3]. The prevalence has been estimated from 3 to 30 percent [4] reaching 15% to 71% in low to middle-income countries [5]. In relation to the literature the prevalence of domestic violence may increase during pregnancy, remains unchanged or decreases [6]. Despite the prominence of the dignity of the woman in the Islamic, there are different types of violence against women [7]. The results of a review study conducted in Iran (2014) with an assessment of 38 articles showed that the prevalence of domestic violence in Iranian pregnant women varies from 19.3% to 94.5% [8].

Domestic violence during pregnancy threatens maternal and fetal health and is a dangerous but preventable factor for many perinatal morbidity and mortality [9]. These complications included isolation, reduce maternal social networks, inadequate pregnancy care, inadequate pregnancy related weight gain, vaginal bleeding, spontaneous abortion, preeclampsia, sexually transmitted infections, stress, reduced quality of life, dissatisfaction of pregnancy, and drug and alcohol abuse. Other complications included stillbirth, premature birth, low birth weight, newborn complications, avoidance of breastfeeding, delayed mother infant bonding, maternal abusive behaviors toward their infants, and children personality complications [10–16].

Women who experience domestic violence often use a variety of mental and behavioral strategies to inhibit or stop the violence [17, 18]. Actually, in the face of increasing violence, mothers often revealed increased activity in protecting themselves and their children. Abused pregnant women confront significant difficulties in keeping themselves and the unborn baby safe [19]. Pregnancy can create a common sense of isolation that may be enhanced by experiences of domestic violence and reduce maternal social networks [13]. Social support provides the chance to discuss stressful experiences, receive support and reduced risk of suffering from domestic violence during pregnancy [20–22].

Coping is an essential strategy for managing domestic violence that includes efforts to manage a problem through the continuous change of intellectual and behavioral efforts [23–25]. In domestic violence situations, coping relates to resilience strategies that victims accomplish to minimize harms and handle the situation [26]. The two main forms of coping strategies are problem focused and emotion focused coping [25, 27]. The process of coping with domestic violence can be understood in relation to the social and cultural background, capacity and access to supportive resources, and the severity of the violence [24].

The capability to inhibit and cope with violence is different for pregnant women compared to non-pregnant population [28, 29] and coping strategies are affected by adolescence and pregnancy [30]. Abused pregnant women may use resilience through problem focused coping, adjusting their motherhood, expand levels of their social support, and seeking help [31]. In spite of the increased risks of domestic violence during pregnancy, many women use their experiences effectively such as resilience [32]. As domestic violence during pregnancy and coping with it has a close association with the social, regional and cultural context, it is essential to collect the associated information in various social and geographical contexts [33], therefore conducting the research in a certain local context can be the most important factor in better clarification and move toward resolving the problem of domestic violence.

Iranian Islamic society is a patriarchal society that highlights men’s authority over women in the family [34–36]. Wife’s obedience to the husband, tolerate husband’s violence and keeping the family are women’s duties and considered honorable [34]. Islam has highlighted the issue of family integrity and deems divorce is a lawful but undesirable and discouraged act [36]. Women are defined by their roles as mothers or wives which expect them to sacrifice and put their husband’s and children’s needs ahead of their own to keep their family from breaking up [35]. In such circumstances, abused pregnant women attempt to adopt various strategies to strengthen their marital relationship and prevent family breakup. In order to achieve a deeper understanding about the strategies for managing domestic violence during pregnancy, it is necessary to develop a qualitative study that reflects maternal behaviors and needs. Different qualitative studies on pregnant women's experiences with domestic violence were conducted in the world [19, 30, 37–39], but no evidence exist to explain the experiences of Iranian pregnant women with domestic violence and their strategies for managing domestic violence during pregnancy. Understanding the experience of domestic violence among these women is essential to design evidence based domestic violence prevention strategies and programs. To the best of our knowledge, this study would be the first one to explore Iranian women's strategies for managing domestic violence during pregnancy.

## Methods

### Design

The conventional content analysis approach was used to design this qualitative study to get deeper insight into pregnant women's experiences of managing domestic violence [40]. Qualitative content analysis is a proper method to study cultural related contextual issue [41] in health science study [42].

### Setting

The current study was carried out from October 2019 to June 2021 in Mashhad, the capital of Khorasan Razavi Province, one of the most populous city in the north east of Iran. At first obstetrics and gynecology departments of teaching hospitals were used to select the participants. The reason for this selection were high referral of pregnant women and effective management of these departments. Purposive sampling led to the selection of comprehensive health center, prenatal clinic, midwifery counseling center, provincial welfare center, forensic medicine center, social emergency, consultant voice center, and other related organization as study setting.

### Data collection

Purposive sampling with a maximum variation such as age, occupational status, education, number of marriages, gestational age, wanted or unwanted pregnancy and domestic violence screening score was used to select the participants. Data were collected through individual semi structured in-depth interviews with 13 women who experienced perinatal domestic violence, one husband and one daughter and 24 key informants comprising of health care professionals as well as the specialists in reproductive health, social working, forensic medicine, psychology, law, sociology and media. Two focus group discussions with attendance of 20 abused mothers were conducted because of their resistance to be interviewed individually. The participants' profile is shown in Tables 1 and 2.

**Table 1:** The profile of women participated in the study (Mashhad, Iran, 2019–2021)

Participant	Age of mother/husband	Education of mother /husband	Number of marriage mother/husband	Duration of marriage	Mother's job	Husband's job	Number of children	Gestational age	Wanted/unwanted pregnancy	Economic status	HITS score	Interview duration
1	37/53	8 years/8 years	1st/2nd	8 years	House wife	Retired	1	37w	Unwanted	Fairly appropriate	16	56 m
2	22/29	7 years/diploma	1st/1st	3 years	House wife	Factory worker	0	39w	Wanted	Good	12	50 m
3	29/30	7 years/diploma	1st/1st	9 years	House wife	Unemployed	1	40w	Wanted	Poor	11	40 m
4	19/30	Diploma/Associate Degree	1st/1st	4 years	House wife	Factory worker	0	35w	Wanted	Good	17	105 m
5	25/25	Illiterate/6 years	1st/1st	3 years	House wife	Factory worker	1	10 h postpartum	Unwanted	Fairly appropriate	12	40 m
6	41/47	Diploma/6 years	1st/1st	25 years	House wife	Sales Manager	4	45 days postpartum	Unwanted	Good	16	90 m
7	36/31	Diploma/6 years	2nd/2nd	5 years	House wife	Driver	2 mother/1 husband	17w	Unwanted	Poor	18	30 m
8	24/28	Diploma/diploma	1st/1st	5 years	House wife	Factory worker	2	20w	Unwanted	Good	14	50 m
9	28/26	Diploma/6 years	2nd/2nd	5 years	Employed	Private business	1	8w	Unwanted	Good	20	95 m
10	36/35	Doctorate/master degree	1st/1st	8 years	Factory production manager	Factory production manager	1	1 year after delivery	Wanted	Good	12	120 m
11	36/40	Master degree/diploma	1st/1st	12 years	Engineer	Self employed	1	1 year after delivery	Wanted	Good	12	80 m
12	26/31	Master degree/doctorate	1st/1st	4 years	University teacher	Doctor	0	39w	Wanted	Good	12	65 m
13	36/40	Bachelor degree	1st/1st	4 years	Employer	Self employed	1	1 year postpartum	Unwanted	Good	12	60 m

Participant	Age	Education	Job	Family relationship	Interview duration
14	22	Bachelor degree	No job	Daughter	30
15	30	Diploma	Employee	Husband	75

**Table 2:** The profile of the Key informants (Mashhad, Iran, 2019–2021)

Participant	Age	Education	Field of study	Work experience (years)	Job position	Interview duration (min)
16	48	Bachelor’s degree	Midwifery education	23	Responsible midwife of health base	115
17	35	Bachelor degree	Counseling	12	Social Emergency Supervisor	40
18	52	Master’s degree	Sociology	25	Expert in charge of social welfare in the province	75
19	45	Master's degree	Counseling	10	Consultant voice and In-person counseling center supervisor	75
20	50	Specialty in Medicine	Forensic medicine	15	Forensic expert	75
21	53	Bachelor’s degree	Midwifery education	32	Retired maternity staff and founder of midwifery counseling office	65
22	48	Master’s degree	Midwifery education	26	Responsible midwife, Midwife of the midwifery clinic	75
23	35	Doctor of Philosophy	Health Psychology	10	Psychiatrist of the Comprehensive Health Center	120
24	35	Bachelor’s degree	Sociology	12	Sociologist and women's activist	55
25	44	Doctor of Philosophy	Rights	20	One of the provincial justice officials	35
26	38	Master’s degree	Criminal Law and Criminology	15	Lawyer and women's activist	60
27	37	Doctor of Philosophy	Reproductive health	14	University Assistant Professor	85
28	48	Doctor of Philosophy	Psychology	20	psychologist of pre-divorce counseling team	75
29	42	Master’s degree	Psychology	12	Social worker of pre-divorce counseling team	40
30	47	Master’s degree	Midwifery education	25	Head of the Midwifery Department of the University of Medical Sciences	45
31	47	Bachelor’s degree	Social work	23	Hospital social worker	55
32	51	Bachelor’s degree	Social service	22	Head of the University Social Welfare Unit	40
33	37	Master’s degree	Midwifery education	12	University Instructor	75
34	45	Master’s degree	Maternal and child health	20	Responsible midwife	60
35	45	Bachelor’s degree	Midwifery education	23	Maternity ward	60
36	48	Master’s degree	Maternal and child health	25	Director of the midwifery department, University instructor	65
37	46	Bachelor’s degree	Midwifery education	11	Expert of the provincial health center	45
38	48	Master’s degree	Nursing	22	Deputy of one of the branches of the Relief Committee	30
39	51	Doctor of Philosophy	Medical ethics	26	University professor, media activist and director of various national media programs	45

Inclusion criteria were maternal agreement for participation in the study, their ability to communicate with the researcher, endorsing perinatal domestic violence and ability to share their related experiences. Exclusion criterion was maternal physical and mental disease that prevent their participation in the study.

Interviews were conducted by the first author with good experience of qualitative research and 23 years working experience in the field of reproductive health and midwifery. The interviews were accomplished at a time and place that was appropriate to participants and were audio recorded. The interviews continued until the data saturation. Data saturation was obtained following 37 interviews and 2 focus group discussions. However, to confirm data saturation, two further interviews were carried out which revealed no new data in the field of different maternal strategies in managing domestic violence. An interview guide with open ended questions was used to explore participants' experiences as follows “Please describe your experience of violence during pregnancy or postpartum?” Other questions followed the main question were "How do you react when you are abused?", "How does pregnancy influence the managing skills you used?", "What are some of the ways you keep yourself and unborn baby safe?", and "what is your advice for other pregnant abused women and service providers?".

The interviews lasted between 30 and 120 min (mean: 65 min) and each focus group took approximately 90 min.

### Data analysis

Conventional content analysis was used to explain the research question. The data were analyzed concurrently with data collection, based on the Graneheim and Lundman method by MAXQDA software (version 10, VERBI Software, Berlin, Germany) [42]. After each interview, the first author listened to it several times to get an overall perception of the content, and then transcribed the interview verbatim and read it several times to obtain a general understanding of the data. The text of each interview was divided into meaning units as words, sentences, and paragraphs. The meaning units were condensed and condensed meaning units were summarized and coded. Codes were compared based on the similarities and differences and classified into subcategories and categories reflecting the apparent content of the text. Finally the themes were identified showing the concealed concept of the text.

### Trustworthiness

To confirm the rigor and the trustworthiness of the data, Guba and Lincoln's criteria, including credibility, confirmability, dependability and transferability, were applied [43]. To maintain the credibility of the results, the data analysis were reviewed and approved by the participants and three expert qualitative researchers. To increase the credibility purposive sampling with maximum variation and appropriate size was used. For dependability the correctness of data analysis was approved by three external skilled researchers in qualitative research. In order to maintain confirmability some of the transcripts, along with the codes and categories were provided to the main supervisor as well as three other faculty members outside the field of study (all experienced in qualitative research) and the process of analysis was confirmed. To make transferability possible, the research characteristics, including the participants and the context of the study were described in detail, therefore other researchers could be able to evaluate the transferability of the results.

## Results

The abused mothers' age were ranged between 19 and 41 years. Maternal educational levels ranged from illiterate to Doctor of Philosophy degrees. Domestic violence screening score ranged from 11 to 20.

In present study 1568 codes, 20 subcategories, 6 categories and two main themes emerged from the data analysis. Through the data analysis "escape strategies" and "situation improvement strategies" were emerged as the main themes. Escape strategies was comprised of three categories including concealment, passive dysfunctional behaviors and neutral passive behaviors. Situation improvement strategies was comprised of three categories including positively active self-regulation, protecting family privacy and help seeking (Table 2). A more precise presentation of the results is given below. Quotations from the participants are included to better clarified women's strategies for managing domestic violence during pregnancy (Table 3).

**Table 3:** Women's strategies for managing domestic violence during pregnancy (Mashhad, Iran, 2019–2021)

Sub category	Category	Theme
Concealment of violence	Concealment	Escape strategies
Emotional release	Passive dysfunctional behaviors	
Retaliatory behaviors		
Abuse to husband and child		
Helplessness and confusion		
Recourse to divorce		
Placating strategies	Neutral behaviors	
Diverting attention		
Self-actualization	Active self-regulation	Situation improvement strategies
Comprehensive self-care skills		
Promoting positive self-concepts		
Resilience		
Strengthening spirituality		
Constructive purposeful efforts	Protecting family privacy	
Supportive efforts		
Maintaining maternal commitment		
Preserving marriage		
Avoiding social judgments		
Disclosure of violence	Help seeking	
Looking for network support		

## Escape strategies

Abused mothers were constantly confronted with the harms and threats posed by violence and had to deal with these harms in different ways. At first, they tried to reduce the psychological stress of violence in various ways, such as concealment, passive dysfunctional behaviors and neutral behaviors without directly paying attention to husband's violence reduction.

### Concealment

The special socio-cultural conditions of Iran and various contextual factors caused abused mothers to conceal violence.

#### Concealment of violence

Abused mothers were initially at a crossroads in choosing to disclose domestic violence or conceal violence. Mostly abused Iranian mothers concealed perinatal domestic violence despite the routine screening for domestic violence during prenatal care. They hided violence from family, friends, the health care system, and the legal community and in this manner they manage perinatal violence in their ways. One participant stated:

*"In my opinion, all pregnant mothers are abused in some way, but they do not disclose it, they don’t tell this situation to anyone. They hide violence." (Participant 10–36 years old- 1 year postpartum)*

### Passive dysfunctional behaviors

Abused mothers knew they were being harmed by violence, but remained in a relationship due to maternal commitment and lack of support. Usually they used passive dysfunctional behaviors to decrease psychological complication.

#### Emotional release

Abused mothers initially react to their husbands' violence by crying, getting angry, and even laughing. This quote reflects it:

*"When he beats me I shout but he beats more. I cry so much. I can do nothing." (Participant 1–37 years old- 36 weeks of gestation)*

#### Retaliatory behaviors

Some abused pregnant women utilized retaliation and defiance as emotion focused strategies for dealing with domestic violence. Failure to comply with husband's requests, not talking, self-defense, immorality, fighting back were different ways to deal with violence, which had no effect on reducing violence and sometimes intensified spouse's violence. The following quote reflects this:

*"When my husband abuses me, I retaliate. He wants to hit me in the abdomen, but I defend myself and fighting back." (Participant 7–36 years old- 17 weeks of gestation)*

#### Abuse to husband and child

Some abused pregnant women try to get rid of psychological distress by sexual disobedience, sexual ignorance and even extramarital relationships and thoughts of killing their husbands. One participant stated:

*"I got very upset when my husband bothered me so I slept in another room and I did not allow him to have sex." (Participant 7–36 years old- 17 weeks of gestation)*

Under the psychological stress of perinatal domestic violence a few mothers hit the fetus, harassed and beat their children, and even left them. One participant declared:

*"When I was angry because of my husband's violent behavior, I hit the baby in my stomach and empty myself in such a way." (Participant 5–25 years old- 10 h postpartum)*

#### Helplessness and confusion

Pregnancy and postpartum create a sense of isolation for mother that magnified by experiences of domestic violence. Inefficient self-talk, leave spirituality, inattention to herself, self-worthlessness, self-forgetfulness and wish for death were maternal strategy for dealing with the emotional effect of perinatal domestic violence. One participant explained:

*"I have not prayed for a long time. Previously, nothing could stop me from praying. But I stopped praying for a while now. I'm very sad. Every time I did not pray, I felt as if I lost something. I lost my way." (Participant 2–22 years old- 39 weeks of gestation)*

#### Recourse to divorce

A few abused mothers decided to divorce as the final solution to get rid of their abusive husband when they could not find another way to reduce the violence. One mother said:

*"If my husband's behavior had not changed and he continued to be violent, I would have separated from him, even with a child, because my child would be under more pressure in this stressful life." (Participant 11–36 years old- 1 year postpartum)*

### Neutral behaviors

The result of the present study indicated that neutral passive behavior such as placating strategies and diverting attention were of the common strategies used by abused mothers to minimize the recurrence and intensifying violence in their marital relationships.

#### Placating strategies

Placating and conscious acceptance of violence were recommended by most of participants as one of the best ways of minimizing the recurrence of perinatal domestic violence. Placating such as silence, tolerance, patience, obedience, indifference, waiver and violence normalization were of the best ways of keeping peace in the house. The following quote reflects it:

*"A pregnant woman may face the worst insults, disrespects and behaviors in her husband's house, but she must be silent, she should be quiet and calm in the house." (Focus group discussion- 35 years old- 1 year postpartum)*

#### Diverting attention

Diverting attention from the issue of violence, forgetting and justification mechanism were maternal strategies for dealing with domestic violence. The following statement confirm this:

*"Despite violent behaviors of my husband, I tried to calm down by diverting my attention, I was involved with my baby in my womb. This made me not to think about violence." (Participant 2–22 years old- 39 weeks of gestation)*

## Applying situation improvement strategies

Some mothers, while believing in the necessity of covering up violence with regard to individual decision-making and individual abilities, and in the shadow of high self-esteem and self-confidence or disclosing violence and seeking support tried to improve their situation by active self-regulation, protecting family privacy and help seeking.

### Active self-regulation

Maternal urge to protect the unborn baby and protect marital life were of the most important impetus to reduce domestic violence by self-actualization, comprehensive self-care skills, promoting positive self-concepts, resilience and strengthening spirituality.

#### Self-actualization

Some abused mothers used the maternal active strategies for dealing with domestic violence in order to be able to protect the privacy of the marital life without causing psychological harm. These strategies included creating a good mood, self-relaxation, return attention through enjoyable activities, positive mental imagery and maintain authority, skills and empowerment. These quote reflects it:

*"Every time my husband was beating me, the baby in my womb was in a bad mood, her movements were slowing down, but I was entertaining myself. Every day, as soon as my husband left the house, I would do the housework and go for a walk with my children to calm down so that we would feel better. "(Participant 6–41 years old- 1 month postpartum)*

#### Comprehensive self-care skills

The abused mothers acquired comprehensive self-care skills through physical self-care, emotional psychological self-care, social self-care and spiritual self-care. The following statement approve this:

*"I was prioritizing for myself. I made time for myself. I even went to the park so that the baby in my womb could hear the sound of the babies in the park. I controlled myself, I calmed myself down." (Participant 6–41 years old- 1 month postpartum)*

#### Promoting positive self-concepts

Maintaining and promoting self-confidence, self-esteem and self-control were the strategies that the abused mothers used to promote positive self-concepts. The following statement confirm this:

*"Some pregnant women lost their self-confidence during pregnancy. They think that their husbands are no longer interested in them, because their appearance has become ugly and they have become fat so their situation is getting worse day by day. I never told my husband that I was ugly during my pregnancy and postpartum. I always told I 'm very good and I have no problems." (Participant 11–36 years old- 1 year postpartum)*

#### Resilience

Despite the increased risk of domestic violence during pregnancy, many women effectively navigate their experiences and come to display adaptive outcomes, such as resilience. Resilience included promote individual growth, being purposeful, internal control, flexibility, realistic look and thinking positive. One participant stated:

*"I think a part of the husband's violence is because of economic issues. I understand the pressure on my husband in life. I think it is fair to tolerate verbal violence occasionally, but not other types of physical, sexual and emotional violence, especially during pregnancy." (Focus group discussion- 38 years old- 6 months postpartum)*

#### Strengthening spirituality

Abused mothers endorsed strengthen spirituality as a form of adaptive coping strategy in dealing with perinatal domestic violence. Relying on God, appealing to the Imams and submitting to the divine destinies were the strategies which resulted in maintaining maternal peace and reducing her vulnerability and ultimately increasing maternal ability to protect the privacy of the marital life. This quote reflects it:

*"No one can help me. Only God can help me. Only God can create an opening in my life and improve my situation. Only Imam Reza can help me." (Participant 3–29 years old- 40 weeks of gestation)*

### Protecting family privacy

The result of the present study showed that protecting family privacy by a purposeful effort to correct spouse behavior, supportive efforts and maintain maternal commitment were the active maternal problem solving strategy for dealing with domestic violence.

#### Constructive purposeful efforts

In the initial exposure to violence, some mothers tried to control violence by a purposeful effort to correct husband's behavior such as turn disputes into positive negotiation, building trust, create intimacy, promote his information, maintain his authority, meet his sexual needs, encourage him for psychological counseling and trying to change his destructive behavior. The following statements indicate it:

*"I tried to maintain my husband's authority and I did not do anything without his information. When the fetal movement was reduced, I endured a lot of anxiety from morning to night, but I didn't want to go to the hospital without my husband's information to control the fetal heart rate." (Participant 11–36 years old- 1 year postpartum)*

#### Supportive efforts

Some mothers supported their husbands in economic and emotional status. Although supportive efforts did not reduce the husband's violence, it could improve the mother's psychological states and it affected the mother's sense of satisfaction with her ability and empowerment. One educated mother stated:

*"I was teaching at university before I got pregnant, I had some savings. I paid for my prenatal care and screening tests because I felt my husband was resisting paying for them. I was so happy to be able to pay for my prenatal care." (Participant 10–36 years old- 1 year postpartum)*

#### Maintaining maternal commitment

Maternal commitment such as efforts to maintain fetal health, trying to keep the children calm and priority the comfort of the fetus and children to maternal liberation from violence made mother to stay in an abusive relationship and didn't think about separation. In fact, mothers sacrificed themselves and put their children’s needs ahead of their own to keep their family from breaking up. One mother explained:

*"When you become a mother, the feeling of motherhood makes you look at life differently, you are responsible for maintaining the health of your child, and the most important thing is to be patient and tolerant." (Participant 1–37 years old- 36 weeks of gestation)*

#### Preserving marriage

Some abused mothers tried to save their marital life despite their husbands' violence because of their interest in cohabitation and their husband's satisfactory commitment. One participant stated:

*"Although my husband sometimes abuses me, but my marital life is good. I think my life is better than my sister's and my mom's. I love my husband. My husband is a man who has been standing on his own feet since he was 15 years old. I 'm very satisfied with my life, I do not want to lose my life." (Participant 5–25 years old- 10 hours postpartum)*

#### Avoiding social judgments

Avoiding social isolation and preventing the stigma of divorce, the stigma of remarriage, preventing the lack of financial support after divorce and the lack of a supportive family prompted the abused mother to try to protect the privacy of the marital life. One participant described:

*"I was born and raised in a small town. If a woman divorces, society and her family look at her with pity and her life will destroy. These are the things that make a mother stay under violent circumstances and try to save her common life." (Focus group discussion- 28 years old- 35 weeks of gestation)*

### Help seeking

The result of the study revealed that some abused mothers finally tried to generate solutions by disclosure violence and gain formal and informal support.

#### Disclosure of violence

Some abused mothers finally disclosed domestic violence following the failure of previous strategies and the intensification of violence. Maternal empowerment and familiarity with individual rights, the ability of the health care system to identify violence, the presence of supportive families and support systems and the intensification of violence have been factors that facilitated the disclosure of violence. The following statements confirm this:

*"Men should know that they have no right to abuse pregnant women. Now I have come to forensics, I want to make it clear to my husband that he has no right to beat a pregnant woman." (Participant 9–28 years old- 8 weeks of gestation)*

#### Looking for network support

Domestic violence reduced maternal social network and the result of the present study demonstrated the importance of formal and informal support and seek help from these support systems in facilitating dealing with domestic violence. Formal support was included health system, psychological counseling, use of welfare services, forensic medical center, social worker, relief committee, the police and Justice. Informal support was included friends and family. Abused mothers formed a friendly alliance and asked help from formal and informal support systems. The following statements confirm this:

*"Abused mothers should be informed about support systems. What services can they receive from the health centers? Where can they go for psychological counseling?" (Participant 27–37 years old- key informant).*

## Discussion

This is the first qualitative study in Iran which identified maternal strategies for managing perinatal domestic violence. According to the results, maternal strategies used for managing perinatal domestic violence included escape strategies and situation improvement strategies. In the shadow of maternal commitment to preserve marriage and child health, mostly abused pregnant women in the early stage of confronting with violence used emotion oriented strategies such as escape strategies for managing violence, gradually the recurrence and intensifying of violence caused applying situation improvement strategies. These results are similar to Lazarus and Folkman conceptualization in which emotion focused coping was used to reduce distress associated with problems such as violence and problem focused coping was used to manage the problems [44].

Despite the routine screening for domestic violence during prenatal care, the majority of abused Iranian mothers concealed perinatal domestic violence. They hided violence from family, friends, the health care system and the legal system. Several factors were important in concealing violence, such as inadequate information on violence, maintaining social reputation, protecting the unborn child, facing multiple fears, social judgments, lack of family support and poor performance of health care and judicial systems. This result is consistent with Damra et al. [45] study.

Similar to a study by Kaye et al. [30], findings from this study showed abused young mothers adopted retaliation and fighting back as an emotion oriented strategy which usually had no effect on reducing violence but sometimes intensified husband's violence. Similarly, few abused mothers harmed themselves to cope with the psychological stress and presented additional health concerns which is aligned with previous study conducted by Bhandari et al. [19]. Similar to a research by Zakar et al. [46], findings from this study revealed that some abused mothers did not submit demands of their husband, ignored them and used husband abusive behavior such as sexual disobedience to get rid of their psychological distress. These strategies had no effect on reducing violence and sometimes cause more violent behavior of husbands. Domestic violence usually causes maternal social and physical withdrawal to decrease their emotional distress. This result is aligned with the previous study [30].

Consistent with a study conducted by Bhandari et al. [47], findings from the present study indicated that some abused mothers used placating strategies and diverting attention to minimizing the recurrence of perinatal domestic violence and keeping peace in the their house.

Some abused mothers used active self-regulation such as self-actualization, comprehensive self-care skills, resilience, and strengthening spirituality. They could control their distress by these effective strategies. These results indicate the importance of educating mothers about the cycle of domestic violence, the warning signs to look for in marriage and how to develop safety after termination of a violent marital life that is consistent with another study [48]. Self-care is recognized mindful activities and taken by women, families and societies to promote the health status [49].

Some abused pregnant women displayed resilience and actively responding to their husband's harassment behavior for dealing with domestic violence. Resilience cause maternal wellbeing after violence. The result is similar to the study conducted by Levesque et al. [31] that victimized pregnant women may display resilience.

Consistent with the study conducted by Zakar et al. [46], participants in the current study used strengthening spirituality as a form of emotion oriented strategies. These strategies made abused mother calm in dealing with domestic violence.

Some mothers used family protection strategies and stayed in a marital abusive relationship. Maternal commitment and protection of the fetus were the important motivation for dealing with perinatal domestic violence. This result is in contrast to Bhandari et al. [47] study. Abused mothers in the face of intensifying violence increased activity in protecting themselves and their children, which is similar to the result of previous study conducted by Gillum et al. [18].

Pregnant and postpartum women used safety planning, resisting, placating, and formal and informal support networks to deal with violence in their lives. This result is align with Bhandari et al. [47] study.

Social support was associated with reduced risk of perinatal domestic violence since this kind of formal support could provide the opportunity for abused mothers to discuss about their vulnerable experiences and receive support in the context of perinatal domestic violence. This result is consistent with the previous studies [20–22].

Family support could help abused mothers to reduce violence and promoting maternal and fetal health. Similarly, family support could help mothers decide to leave the abused marital relationship which is consistent with previous studies [25, 50].

Women who received psychosocial counselling showed a reduction in the frequency and severity of domestic violence, and this included both physical and emotional abuse. Psychology counseling could be an effective approach to reduce the recurrence of violence in pregnant women exposed to domestic violence. The basics of counseling was empowerment and increasing self-condense. Through this way abused mothers found that they are able to find appropriate solutions to solve domestic violence through their empowerment and positive negotiations with their husbands. The results were similar to the previous studies [51, 52]. The result of the present study recommended that life skills training classes should be considered for abused pregnant women in health centers to reduce the prevalence of prenatal domestic violence which was similar to the study conducted by Taghizadeh et al. [53].

In line with the study conducted by Zakar et al. [46], Bhandari et al. [47] and Kaye et al. [30] some abused mothers used problem solving strategies such as gaining formal and informal network support and asked help from these support systems which could facilitate dealing with violence.

The results of this study increase understanding of Iranian pregnant women's strategies for managing perinatal domestic violence which could enable support systems to encourage abused mothers to use more effective strategies and seeking help to reduce domestic violence. These include strengthening formal and informal support systems and facilitating access for abused pregnant and postpartum women to formal and informal services, particularly the effective health care system.

According to the different cultural values of Iranian society further research is needed to explore facilitators and barriers of disclosing domestic violence during pregnancy. Similarly, further research is also needed to design effective interventions for women who report domestic violence during and after pregnancy.

## Strength and limitation

This is the first study performed in Iran to explore Iranian women's strategies for managing domestic violence during pregnancy. Similarly, Data collection through interviewing with specialists from various disciplines, diversity of participants and multiple perspectives of domestic violence during pregnancy and postpartum were the strengths of this study. Patriarchal cultural context of Iran and the difficulty to obtain responses from the abused mothers considering the taboo of violence were important limitations of this study. Information regarding domestic violence during pregnancy is considered an individual family issue and should not be discussed with strangers even though they are health care providers that may, in turn, lead to under reporting of domestic violence during pregnancy by the participants.

### Practical implications

Despite the methodological limitations of the study, our findings will be useful to health care providers, program managers, and policy makers in addition to women and human rights activists. They can be used in the development of specialized training and materials for building providers capacities to deal more effectively with cases of domestic violence during pregnancy and postpartum and it is also fundamental to collaborate with other professionals in order to develop shared health-care pathways.

## Conclusion

The high prevalence and the negative consequences of domestic violence against pregnant women provide a golden opportunity for performing interventions. Pregnancy and protection of unborn child could affect maternal strategies for dealing with domestic violence. The results of the various strategies that the mother used to reduce perinatal domestic violence vary from reducing domestic violence to not changing the intensity of the violence or intensifying the perinatal domestic violence. It is noteworthy that adopting inappropriate strategies against domestic violence could be threatening for fetal and maternal life and appropriate strategies should be considered and emphasized in maternal education. Understanding the experience of domestic violence among these women is essential to design evidence based domestic violence prevention strategies and programs.

## Data Availability

The datasets used and/or analyzed during the current study are available from the corresponding author on reasonable requests.
